# Dynamic Changes in Apolipoproteins During Anthracycline Therapy: An Exploratory Study of Potential Biomarkers of Cardiotoxicity in Breast Cancer Patients

**DOI:** 10.3390/jcm15218250

**Published:** 2026-10-23

**Authors:** Beata Puton, Agata Makówka, Anna Borowiec, Patrycja Ozdowska, Agnieszka Maria Żebrowska, Maciej Skrzypczak, Beata Kotowicz

**Affiliations:** 1 Cancer Biomarkers and Cytokines Laboratory Unit, Department of Laboratory Diagnostics, Maria Sklodowska-Curie National Research Institute of Oncology, Roentgena Str. 5, 02-781 Warsaw, Poland; agata.makowka@nio.gov.pl (A.M.); beata.kotowicz@nio.gov.pl (B.K.); 2 Non-Commercial Clinical Research Outpatient Clinic, Maria Sklodowska-Curie National Research Institute of Oncology, Roentgena Str. 5, 02-781 Warsaw, Poland; anna.borowiec@nio.gov.pl (A.B.); agnieszka.zebrowska@nio.gov.pl (A.M.Ż.); 3 Department of Breast Cancer and Reconstructive Surgery, Maria Sklodowska-Curie National Research Institute of Oncology, Roentgena Str. 5, 02-781 Warsaw, Poland; patrycja.ozdowska@nio.gov.pl; 4 Unit for Screening Studies in Inherited Cardiovascular Diseases, The Cardinal Stefan Wyszynski National Institute of Cardiology, 04-628 Warsaw, Poland; 5 Department of Gynecology, Medical University of Lublin, 20-954 Lublin, Poland; skrzypczakmk8@gmail.com

**Keywords:** anthracyclines, cancer therapy-related cardiac dysfunction, apolipoproteins, ApoC3, ApoE, biomarkers, breast cancer, cardio-oncology

## Abstract

**Background/Objectives:** Cancer therapy-related cardiac dysfunction (CTRCD) is a clinically important complication of anthracycline-based chemotherapy, and early identification of patients at increased risk remains challenging. Apolipoproteins involved in lipid metabolism and inflammatory pathways may reflect cardiovascular alterations induced by anticancer treatment. This study aimed to evaluate changes in selected apolipoproteins and C-reactive protein (CRP) during anthracycline therapy and to assess their association with the development of CTRCD in patients with breast cancer. **Methods:** This prospective study included 54 patients with breast cancer treated with anthracycline-based chemotherapy at the Maria Sklodowska-Curie National Research Institute of Oncology in Warsaw, Poland. Plasma concentrations of apolipoproteins A1, A2, B, C1, C3, D, E, H, and J, as well as CRP, were measured using an xMAP multiplex assay at baseline, after completion of chemotherapy, and approximately 6 months after treatment. Cardiac biomarkers, including high-sensitivity cardiac troponin T (hs-cTnT) and NT-proBNP, were also assessed. CTRCD was diagnosed based on standard clinical and echocardiographic criteria. **Results:** Anthracycline therapy significantly affected the apolipoprotein profile, with decreased concentrations of apolipoproteins A1, A2, B, C1, E, H, and J, and increased concentrations of apolipoproteins C3 (ApoC3) and D and CRP during follow-up (all *p* < 0.001). Patients with CTRCD showed higher concentrations of selected apolipoproteins, particularly apolipoproteins C3, E (ApoE) and A2. The change in ApoC3 concentration from baseline to 6 months demonstrated the strongest predictive performance for CTRCD (AUC = 0.875; 95% CI: 0.735–1.000; *p* < 0.001). A cut-off value of ≥31.50 mg/dL provided 83.3% sensitivity and 87.5% specificity. **Conclusions:** Changes in apolipoprotein concentrations, particularly an increase in ApoC3 during anthracycline therapy, may be associated with the development of CTRCD. Assessment of ApoC3 and ApoE after chemotherapy may provide additional information for identifying breast cancer patients at higher risk of cardiac dysfunction.

## 1. Introduction

### 1.1. I. Mechanisms of Anthracycline-Induced Toxicity

Anthracyclines, including doxorubicin and epirubicin, have long been established as a cornerstone of systemic anticancer therapy due to their potent antitumor activity. Their principal mechanism of action involves the inhibition of topoisomerase II, resulting in DNA double-strand breaks, cell cycle arrest, and ultimately apoptosis. In addition, anthracyclines intercalate into DNA, thereby disrupting DNA replication and RNA transcription. They also induce the generation of reactive oxygen species, which contribute to oxidative damage of DNA, proteins, and cellular membranes [1].

Despite the development of novel therapies, anthracycline-based regimens remain a cornerstone in the treatment of breast cancer, particularly in patients at high risk of recurrence [2,3]. However, their activity is not limited to malignant cells. The most clinically significant adverse effect is cardiotoxicity, defined as cancer therapy-related cardiac dysfunction (CTRCD). CTRCD is characterized by impaired left ventricular systolic function associated with cancer therapy, leading to a decrease in left ventricular ejection fraction and/or symptoms of heart failure. Due to the pleiotropic mechanisms of action of anthracyclines, their effects are not limited exclusively to malignant cells. The most clinically significant adverse effect associated with this class of agents is cardiotoxicity, referred to as cancer therapy-related cardiac dysfunction (CTRCD). CTRCD is defined as impairment of myocardial systolic function induced by anticancer therapy, leading to a reduction in left ventricular ejection fraction and/or the development of symptoms of heart failure [4,5].

The mechanisms underlying anthracycline-induced cardiotoxicity have not been fully elucidated. However, the generation of reactive oxygen species and the resulting oxidative stress are considered key contributors, leading to damage of cellular structures and cardiomyocyte death [6,7]. Other proposed mechanisms of cardiomyocyte injury include dysregulation of intracellular signaling pathways, alterations in gene expression, inhibition of ion pump activity resulting in disturbances in iron homeostasis, calcium overload, mitochondrial dysfunction, impaired protein synthesis, and abnormalities in cardiac function [6,8,9].

Cardiotoxicity may initially develop in a subclinical manner, with clinical manifestations such as dyspnea, edema, or symptoms of heart failure becoming apparent only after significant myocardial damage has occurred. Therefore, early detection of subclinical cardiac injury remains one of the major challenges in contemporary cardio-oncology. The identification of reliable biomarkers for the early detection of patients at increased risk of cancer therapy-related cardiac dysfunction (CTRCD) has emerged as an important area of research, as it may enable the implementation of appropriate monitoring and cardioprotective strategies prior to the onset of overt myocardial dysfunction [4,9].

### 1.2. II. Current Methods for Monitoring Cardiotoxicity—Limitations

Current strategies for monitoring cancer therapy-related cardiac dysfunction are primarily based on imaging techniques, particularly echocardiography, and the assessment of circulating cardiac biomarkers such as troponins and natriuretic peptides. However, a growing body of evidence suggests that inflammatory processes and metabolic disturbances also contribute to the pathogenesis of cardiotoxicity.

In particular, C-reactive protein (CRP), a marker of systemic inflammation, has been associated with an increased risk of cardiovascular events and may reflect underlying pathological processes contributing to myocardial injury. Furthermore, early changes in multiple biomarkers, including inflammatory and oxidative stress markers, have been shown to predict subsequent development of cardiac dysfunction in patients undergoing cancer therapy [9,10,11].

In addition, abnormalities in the apolipoprotein profile, particularly the apolipoprotein B/apolipoprotein A1 ratio (ApoB/ApoA1), are associated with an increased risk of vascular disease and reflect a proatherogenic metabolic milieu. Inflammatory processes further contribute to endothelial dysfunction and vascular impairment, linking metabolic and inflammatory pathways in cardiovascular pathology. These mechanisms may enhance the susceptibility of cardiomyocytes to injury during cancer therapy, as highlighted in contemporary cardio-oncology guidelines [4,9,12].

Despite growing interest in novel biomarkers, there are a limited number of studies simultaneously evaluating inflammatory markers and detailed apolipoprotein profiles in the context of cancer therapy-related cardiac dysfunction. While C-reactive protein (CRP) is a well-established and widely used marker of systemic inflammation associated with cardiovascular risk, evidence regarding the role of a broader spectrum of apolipoproteins, such as apolipoprotein B (ApoB) and apolipoprotein A1 (ApoA1), is primarily derived from studies in the general population. This gap is also highlighted in contemporary cardio-oncology literature, which emphasizes the need for improved biomarker-based risk stratification strategies [4,13,14].

A more comprehensive assessment of metabolic and lipid-related pathways may provide additional insights into mechanisms associated with anthracycline-induced cardiotoxicity. Interactions between systemic inflammation, metabolic dysregulation, and cardiovascular function have been increasingly recognized in the context of cancer and its treatment. A better understanding of these processes may contribute to elucidating the mechanisms underlying cardiotoxicity and improving risk stratification in patients undergoing anthracycline-based therapy, as emphasized in contemporary cardio-oncology frameworks [4,6,8].

### 1.3. III. Inflammation and Cardiovascular Risk

Inflammation represents an important component of the pathophysiology of anthracycline-induced cardiotoxicity. Activation of inflammatory pathways, increased levels of pro-inflammatory cytokines, and enhanced cellular stress may contribute to cardiomyocyte injury and endothelial dysfunction.

C-reactive protein (CRP) is a widely used marker of systemic inflammation, and elevated levels have been associated with an increased risk of cardiovascular events. Changes in CRP levels during anthracycline therapy may reflect systemic inflammatory activation and have been associated with the development of cardiotoxicity [15,16].

### 1.4. IV. Apolipoproteins and Cardiovascular Risk

Apolipoproteins constitute the primary protein component of lipoproteins and play a crucial role in lipid metabolism, stabilization of lipoprotein particles, and the transport of cholesterol and triglycerides in plasma. They also function as ligands for cellular receptors and as regulators of enzymes involved in lipoprotein metabolism, thereby influencing lipid homeostasis and the development of cardiovascular diseases [17].

Among the most important apolipoproteins are those associated with high-density lipoproteins (HDL), such as apolipoprotein A1 and apolipoprotein A2 (ApoA2), which are involved in reverse cholesterol transport (RCT) and exhibit protective effects on the cardiovascular system [17,18].

Apolipoprotein A-I, the main protein component of HDL, plays a key role in initiating reverse cholesterol transport by promoting cholesterol efflux from peripheral cells and activating lecithin–cholesterol acyltransferase (LCAT) [19,20]. This process enables the transport of excess cholesterol to the liver for elimination, contributing to its anti-atherosclerotic effects [17].

Moreover, higher concentrations of apoA1 have been shown to correlate with increased cholesterol efflux capacity, further emphasizing its protective role [21].

Apolipoproteins associated with atherogenic lipoproteins also play an important role, such as apolipoprotein B, which is the main component of LDL, VLDL, and IDL particles that are involved in the development of atherosclerotic processes [22,23].

Additionally, apolipoproteins from the C group as well as apolipoprotein E are involved in regulating the metabolism of triglyceride-rich lipoproteins by modulating the activity of lipoprotein lipase and the processes of lipoprotein uptake by the liver [24,25].

Disturbances in apolipoprotein concentrations may reflect alterations in lipid metabolism, inflammatory processes, and endothelial dysfunction, all of which play a significant role in the pathogenesis of cardiovascular diseases [26,27].

Despite growing interest in their clinical relevance, data on changes in apolipoprotein profiles in patients undergoing anthracycline therapy and their association with cardiotoxicity remain limited, with available evidence largely restricted to selected apolipoproteins such as apolipoprotein E (ApoE) and apolipoprotein M and predominantly derived from preclinical or small translational studies [28,29].

This study aimed to investigate the association between circulating levels of selected apolipoproteins and C-reactive protein and the development of anthracycline therapy-related cardiotoxicity (CTRCD) in patients with cancer. Furthermore, we assessed the potential utility of these biomarkers for CTRCD risk stratification and examined their relationship with established markers of myocardial injury and haemodynamic stress, including troponin T and NT-proBNP.

## 2. Materials and Methods

The study included 54 female patients with triple-negative breast cancer treated at the Maria Sklodowska-Curie National Research Institute of Oncology in Warsaw, Poland. The median age of the study population was 62.5 years. The baseline characteristics of the study cohort are presented in Table 1.

The study cohort comprised 54 patients, of whom 41 (76%) had luminal B breast cancer and 13 (24%) had triple-negative breast cancer (TNBC). Patients with luminal B breast cancer received neoadjuvant chemotherapy consisting of four cycles of doxorubicin (60 mg/m^2^ per cycle) in combination with cyclophosphamide, followed by 12 doses of paclitaxel. Patients with TNBC received treatment in the reverse sequence, comprising 12 doses of paclitaxel in combination with carboplatin and pembrolizumab, followed by four cycles of doxorubicin and cyclophosphamide. Two patients (4%) also received trastuzumab. The same doxorubicin dose was administered in both groups (60 mg/m^2^ per cycle for four cycles). All patients subsequently underwent surgery followed by adjuvant radiotherapy. Patients were selected from the study population to form two groups of comparable size based on the development of cancer therapy-related cardiac dysfunction (CTRCD). The study population was intentionally selected to include relatively older patients, given that advanced age is an established risk factor for the development of CTRCD and anthracycline-related cardiotoxicity. The study followed predefined inclusion and exclusion criteria, which included, among others, an ECOG performance status of 0–2, at least moderate baseline cardiovascular toxicity risk according to the anthracycline-specific HFA–ICOS risk stratification [4], preserved left ventricular ejection fraction (LVEF ≥ 50%), and no previous history of heart failure, coronary artery disease, or significant valvular disease. Patients who had previously received chemotherapy or chest radiotherapy were also excluded. Regarding cardiovascular medication use, 30 patients (56%) received statins, 23 patients (43%) received beta-blockers, and 24 patients (44%) received angiotensin-converting enzyme inhibitors (ACE-Is). None of the study participants received liposomal doxorubicin or dexrazoxane.

Blood samples were collected at three predefined time points: before initiation of anthracycline therapy (I), immediately after completion of treatment ((II); mean, 198 days; range, 147–406 days), and approximately 6 months after treatment completion ((III); mean, 182 days; range, 172–196 days).

Samples at time points I and II were collected before the diagnosis of CTRCD, whereas samples at time point III were collected during the period surrounding CTRCD diagnosis. Serum was obtained by centrifugation of venous blood samples and stored at −80 °C until analysis.

Serum concentrations of a comprehensive panel of apolipoproteins, including apolipoproteins A1, A2, B, C1, C3, D, E, H, and J, were determined as lipid biomarkers. Cardiac biomarkers included N-terminal pro-B-type natriuretic peptide (NT-proBNP) and high-sensitivity cardiac troponin T (hs-cTnT), while C-reactive protein (CRP) was assessed as a marker of systemic inflammation.

Serum concentrations of apolipoproteins and CRP were measured using an xMAP multiplex bead-based immunoassay on the Bio-Plex 200 system (Bio-Rad Laboratories, Hercules, CA, USA) with Bio-Plex Pro™ Human Apolipoprotein Assays (Bio-Techne Corporation, Minneapolis, MN, USA). Concentrations of cardiac biomarkers, including NT-proBNP and hs-cTnT, were determined by electrochemiluminescence immunoassay using the cobas pro e 801 analyzer (Roche Diagnostics, Mannheim, Germany) with the Elecsys NT-proBNP and Elecsys Troponin T hs assay kits (Roche Diagnostics, Mannheim, Germany).

Patients were classified into the CTRCD or non-CTRCD group according to whether they developed CTRCD during follow-up after anthracycline exposure. CTRCD was defined as a ≥10-percentage-point decrease in left ventricular ejection fraction (LVEF) to a value below 50% and/or a relative reduction in global longitudinal strain (GLS) of >15%, in accordance with the criteria described in the 2022 ESC cardio-oncology guidelines [30]. Overall, CTRCD was observed in 55.5% of patients in the selected study cohort.

The study was conducted in accordance with the principles of the Declaration of Helsinki. The study protocol was approved by the Independent Ethics Committee of the Maria Sklodowska-Curie National Research Institute of Oncology in Warsaw, Poland (approval date: 28 October 2021; approval No. 80/2021), and all participants provided written informed consent before enrollment. The study was registered at ClinicalTrials.gov (identifier: NCT05118178).

Statistical analyses were performed using jamovi software (version 2.6.44). The distribution of the data was assessed before conducting the analyses. Continuous variables were expressed as medians and interquartile ranges (IQRs). Differences between two independent groups were assessed using the Mann–Whitney U test, whereas changes across three predefined time points were analyzed using the Friedman test, followed by post hoc pairwise comparisons using the Durbin–Conover method. Associations between variables were assessed using Spearman’s rank correlation coefficient. Receiver operating characteristic (ROC) curve analyses were performed to evaluate the discriminatory ability of selected biomarkers, and optimal cut-off values were determined using the Youden index. Multivariable logistic regression was used to assess independent associations with cancer therapy-related cardiac dysfunction (CTRCD). Given the exploratory nature of the study, the statistical analyses were primarily intended to identify potential associations and generate hypotheses for further research.

Generative artificial intelligence (GenAI) tools were used during the preparation of this manuscript to assist with information retrieval, clarification and organization of concepts, and refinement of selected passages. The use of GenAI was limited to providing suggestions and supporting the development and refinement of the authors’ own work.

## 3. Results

### 3.1. Association Between Statin Therapy and Serum Apolipoprotein Concentrations

To evaluate the potential effect of statin therapy on serum apolipoprotein concentrations, baseline levels were compared between patients receiving statins (35% of the study population) and those not treated with statins (65%). This analysis was performed to assess whether statin therapy could act as a potential confounding factor in the evaluation of anthracycline-induced cancer therapy-related cardiac dysfunction.

**Figure 1:**
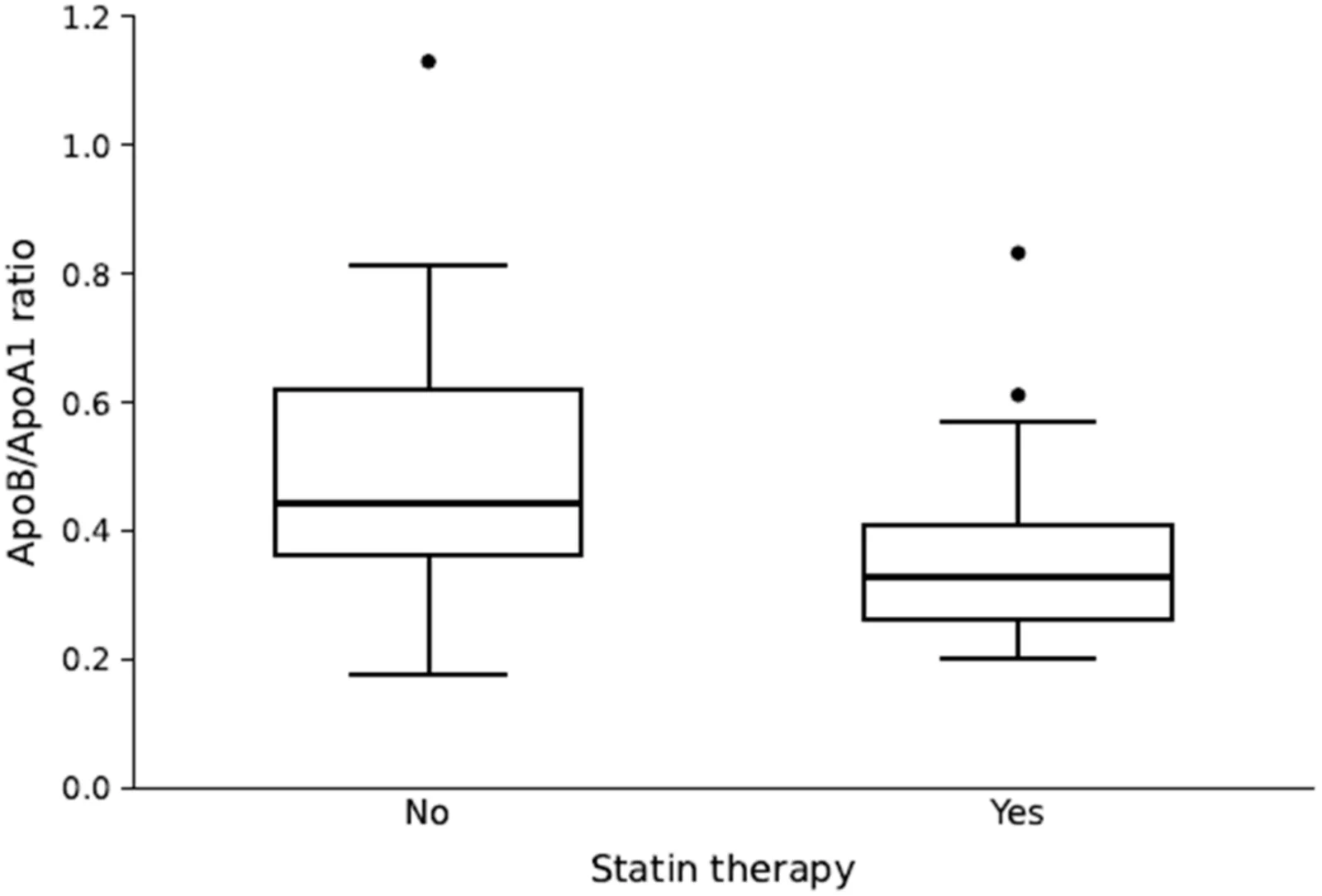
Comparison of the ApoB/ApoA1 ratio between patients receiving statin therapy (Yes) and those not receiving statins (No).

**Figure 2:**
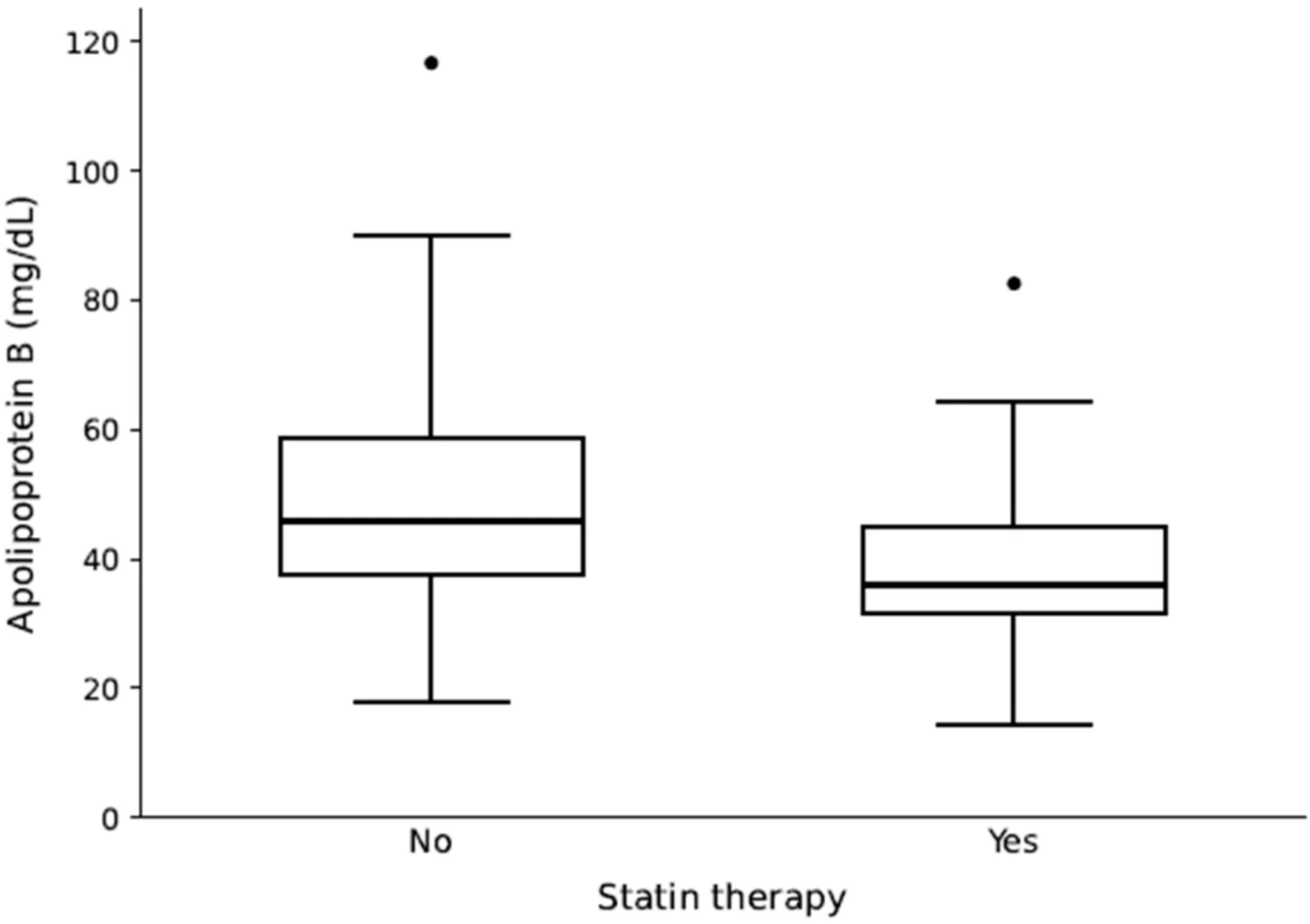
Comparison of apolipoprotein B concentrations between patients receiving statin therapy (Yes) and those not receiving statins (No).

Patients not receiving statin therapy had significantly higher baseline concentrations of apolipoprotein B (U = 198, *p* = 0.015) and a higher ApoB/ApoA1 ratio (U = 190, *p* = 0.009) than patients treated with statins (Figure 1 and Figure 2).

### 3.2. Changes in Biomarker Concentrations Across the Predefined Study Time Points

Analysis of changes in biomarker concentrations across the three predefined time points (before treatment, immediately after treatment, and 6 months after treatment) demonstrated significant differences for all evaluated apolipoproteins and CRP (Friedman test, *p* < 0.001 for all analytes). Post-hoc analysis revealed that these differences were observed between baseline and the 6-month follow-up (time point I vs. III; *p* < 0.001) as well as between the post-treatment assessment and the 6-month follow-up (time point II vs. III; *p* < 0.001). In contrast, no significant differences were found between baseline and the post-treatment measurements (time point I vs. II) (Table 2).

**Figure 3:**
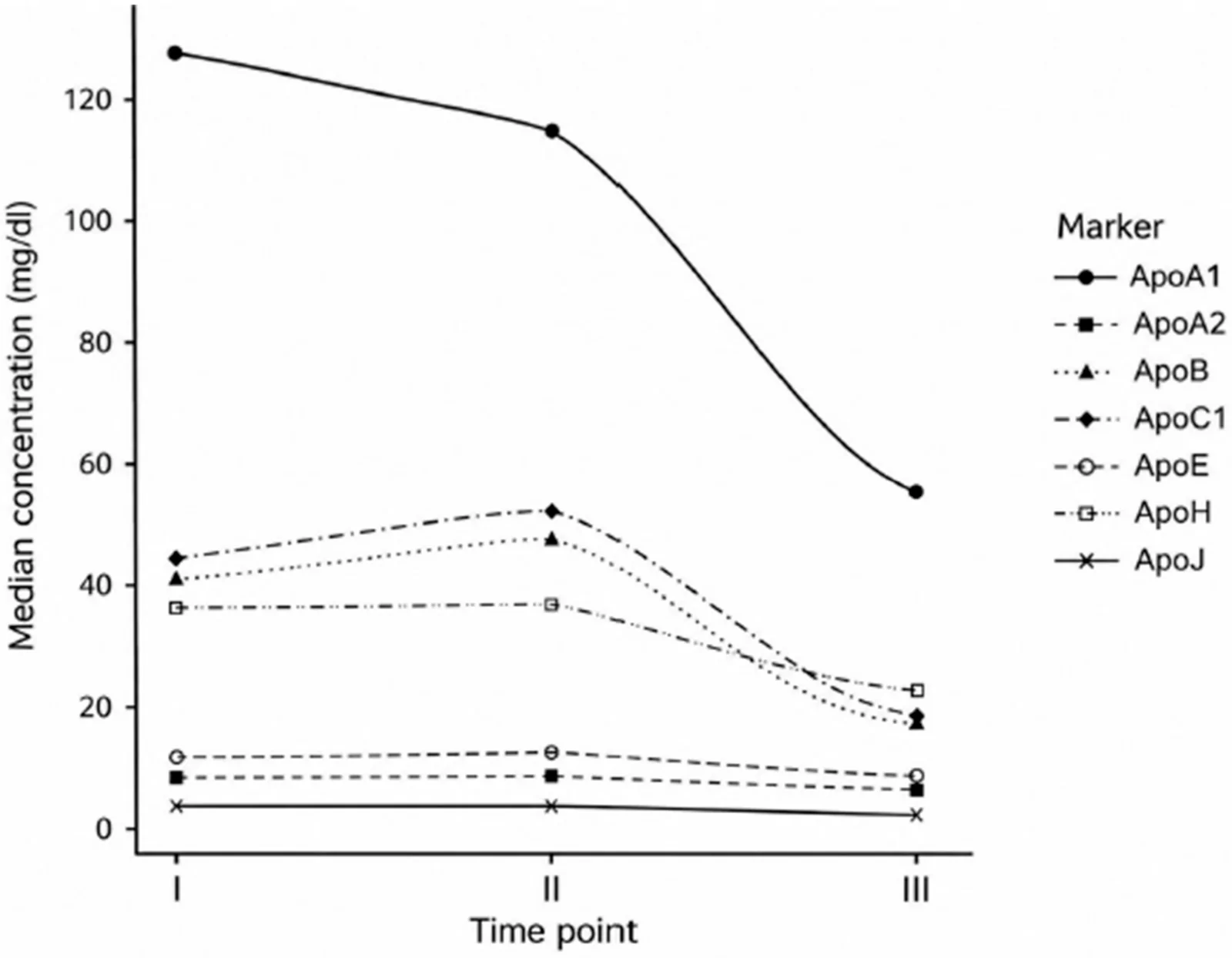
Changes in median concentrations of apolipoproteins A1 (ApoA1), A2 (ApoA2), B (ApoB), C1 (ApoC1), E (ApoE), H (ApoH) and J (ApoJ) across three time points (I–III).

Despite the divergent directions of change, most of the analyzed parameters exhibited a similar temporal pattern. Their concentrations remained largely unchanged between baseline and immediately after treatment completion, whereas significant alterations became apparent only 6 months after the end of therapy. For apolipoproteins A1, A2, B, C1, E, H, and J, this delayed response was characterized by a pronounced decrease in concentrations during the post-treatment follow-up period (Figure 3).

**Figure 4:**
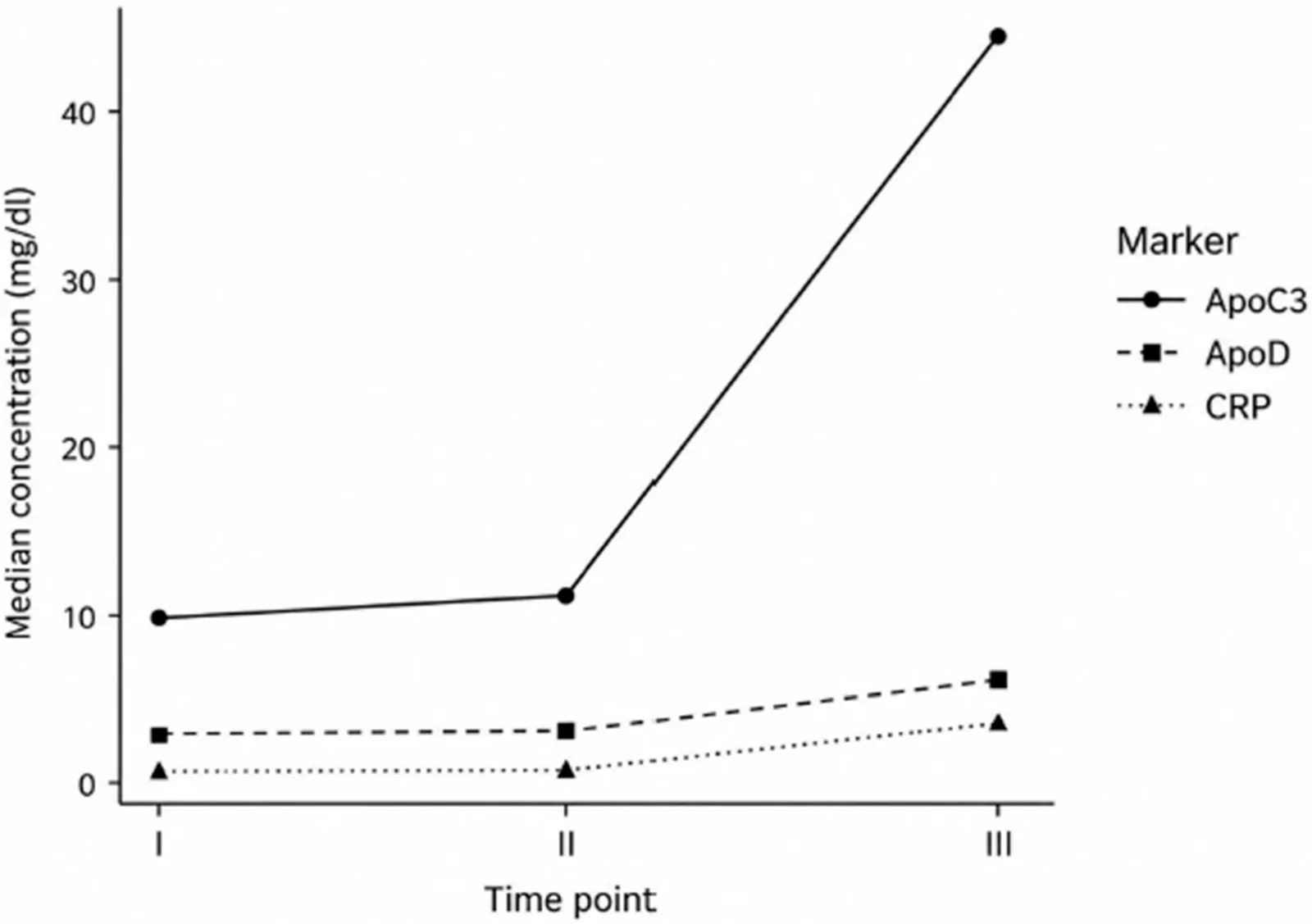
Changes in median concentrations of apolipoprotein C3 (ApoC3), apolipoprotein D (ApoD), and C-reactive protein (CRP) across three time points (I–III).

Conversely, apolipoproteins C3, D, and CRP demonstrated an opposite trajectory, with concentrations remaining stable before treatment and immediately after its completion, followed by a significant increase observed 6 months after the end of therapy (Figure 4).

### 3.3. Assessment of Biomarker Concentrations in Patients with and Without Cardiotoxicity (0 vs. 1)

Significant differences in the concentrations of selected apolipoproteins were observed between patients with and without cardiotoxicity at specific time points of the study.

Differences between the two groups were identified for apolipoproteins A1, A2, C3, D, E, and H. At the 6-month follow-up after treatment completion, patients who developed cardiotoxicity demonstrated significantly higher concentrations of these apolipoproteins compared with patients without cardiotoxicity. Immediately after treatment completion, significant differences between the groups were observed for apolipoproteins C3 and E. At this time point, patients with cardiotoxicity had significantly higher apolipoprotein C3 concentrations and significantly lower apolipoprotein E concentrations compared with patients without cardiotoxicity (Table 3).

**Figure 5:**
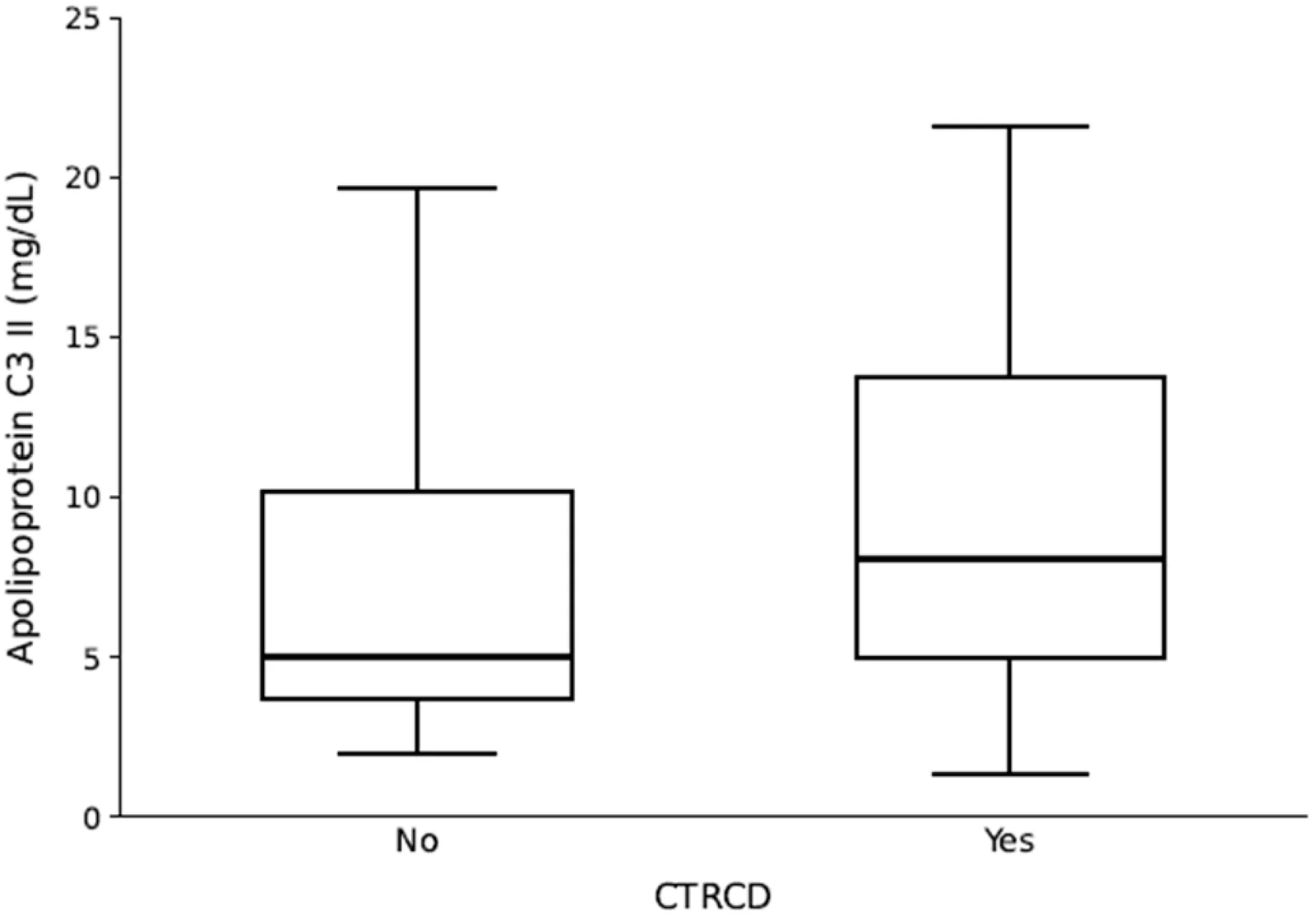
Comparison of apolipoprotein C3 concentrations at time point 2 (Apolipoprotein C3 II) between patients with cardiotoxicity (Yes) and without cardiotoxicity (No).

**Figure 6:**
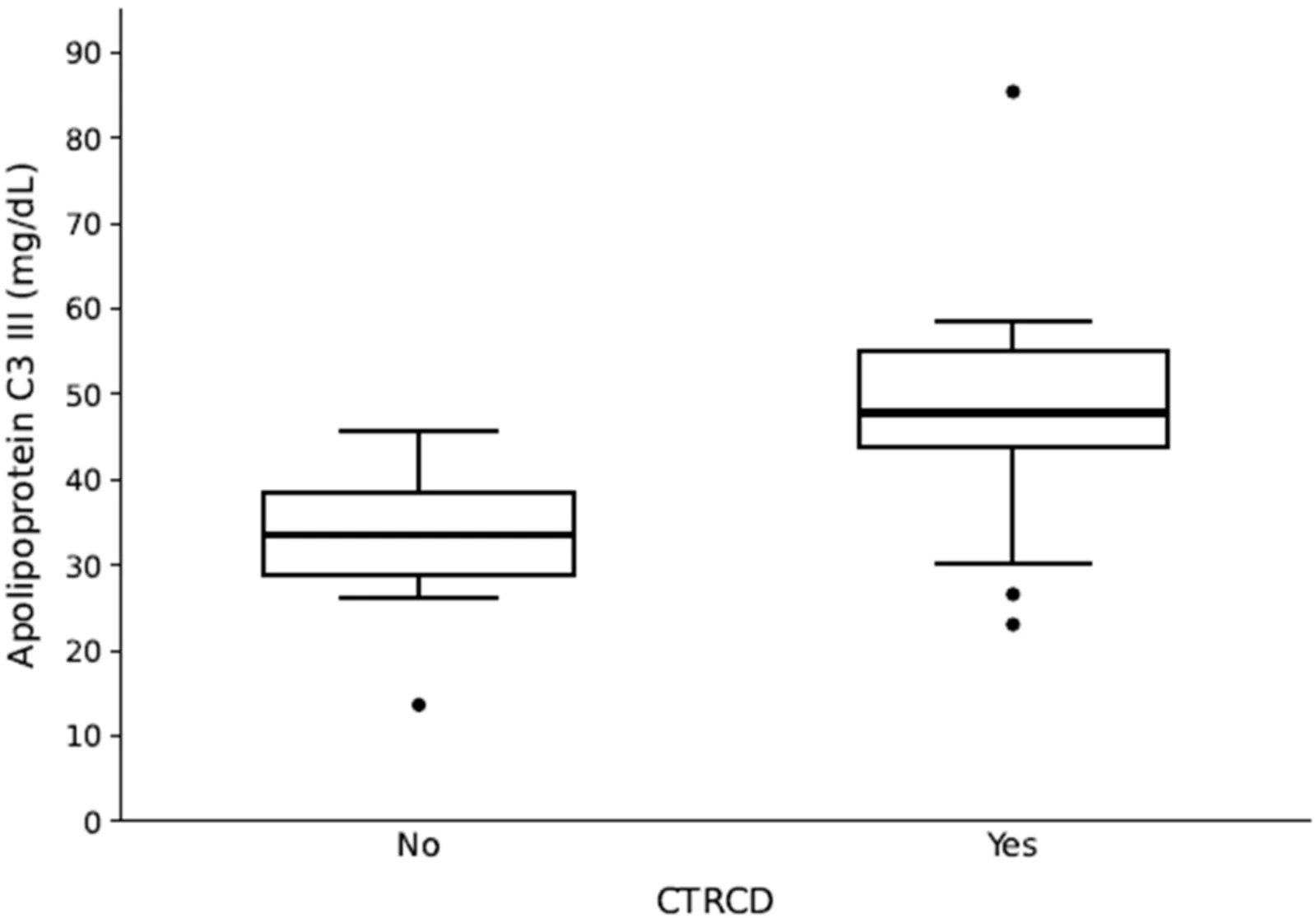
Comparison of apolipoprotein C3 concentrations at time point 3 (Apolipoprotein C3 III) between patients with cardiotoxicity (Yes) and without cardiotoxicity (No).

For apolipoprotein C3 (ApoC3), significant differences between patients with and without cardiotoxicity were observed both immediately after treatment completion (C3 II: 5.00 vs. 8.09 mg/dL; U = 239.0; *p* = 0.036) and at the 6-month follow-up (C3 III: 33.52 vs. 47.82 mg/dL; U = 24.0; *p* = 0.006) (Figure 5 and Figure 6).

**Figure 7:**
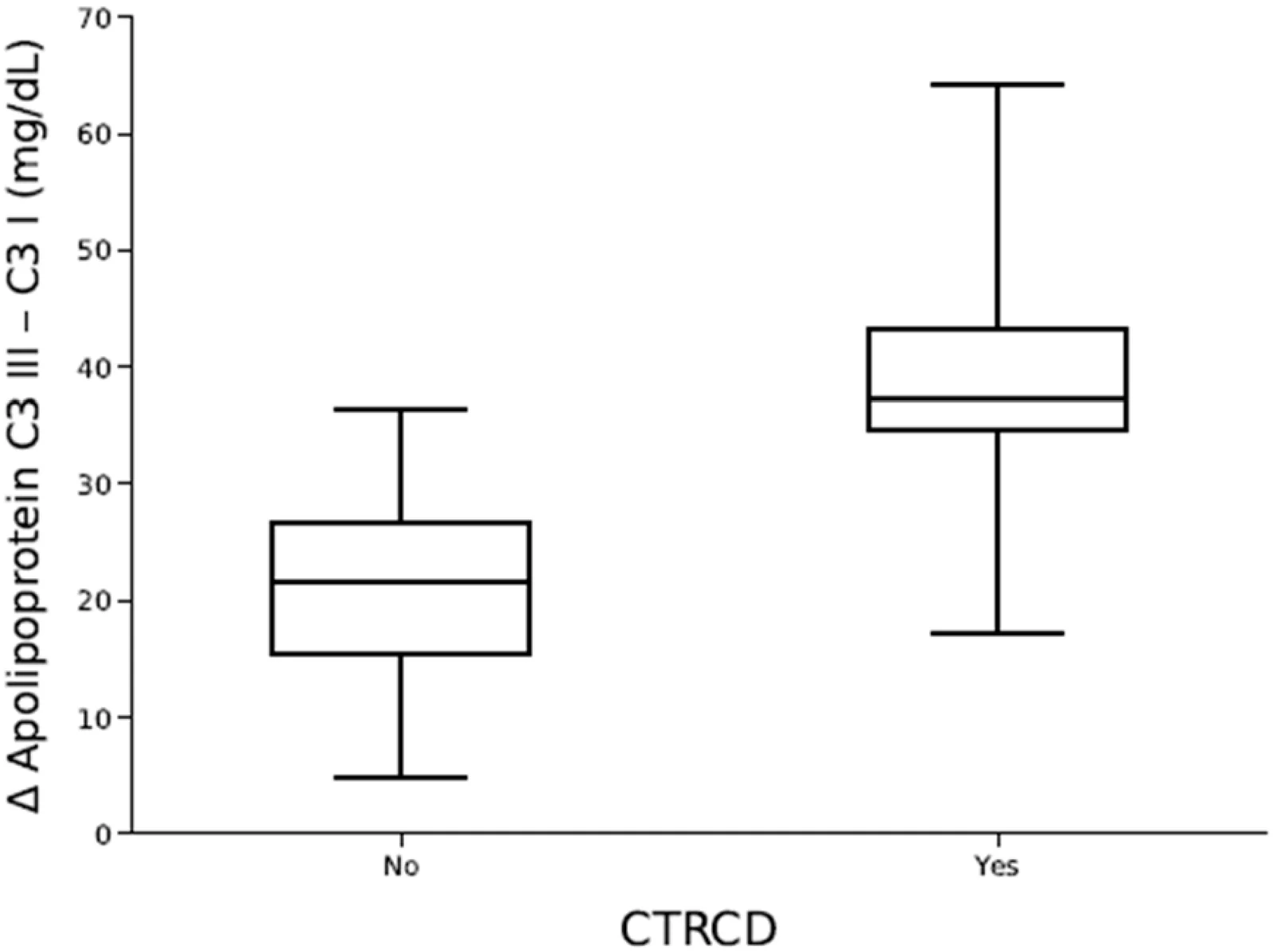
Comparison of the change in apolipoprotein C3 concentrations between time points 3 and 1 (ΔApolipoprotein C3 III–C3 I) in patients with (Yes) and without cardiotoxicity (No).

At both time points, lower ApoC3 concentrations were observed in patients without cardiotoxicity, whereas higher concentrations were found in patients with cardiotoxicity. Furthermore, a significant difference in the change in ApoC3 concentration between baseline and the 6-month follow-up was identified (ΔC3 III–I: 21.52 vs. 37.24; U = 18.0; *p* = 0.002), indicating a significantly greater increase in ApoC3 concentration in patients with cardiotoxicity and a distinct temporal pattern of ApoC3 changes between the analyzed groups (Figure 7).

**Figure 8:**
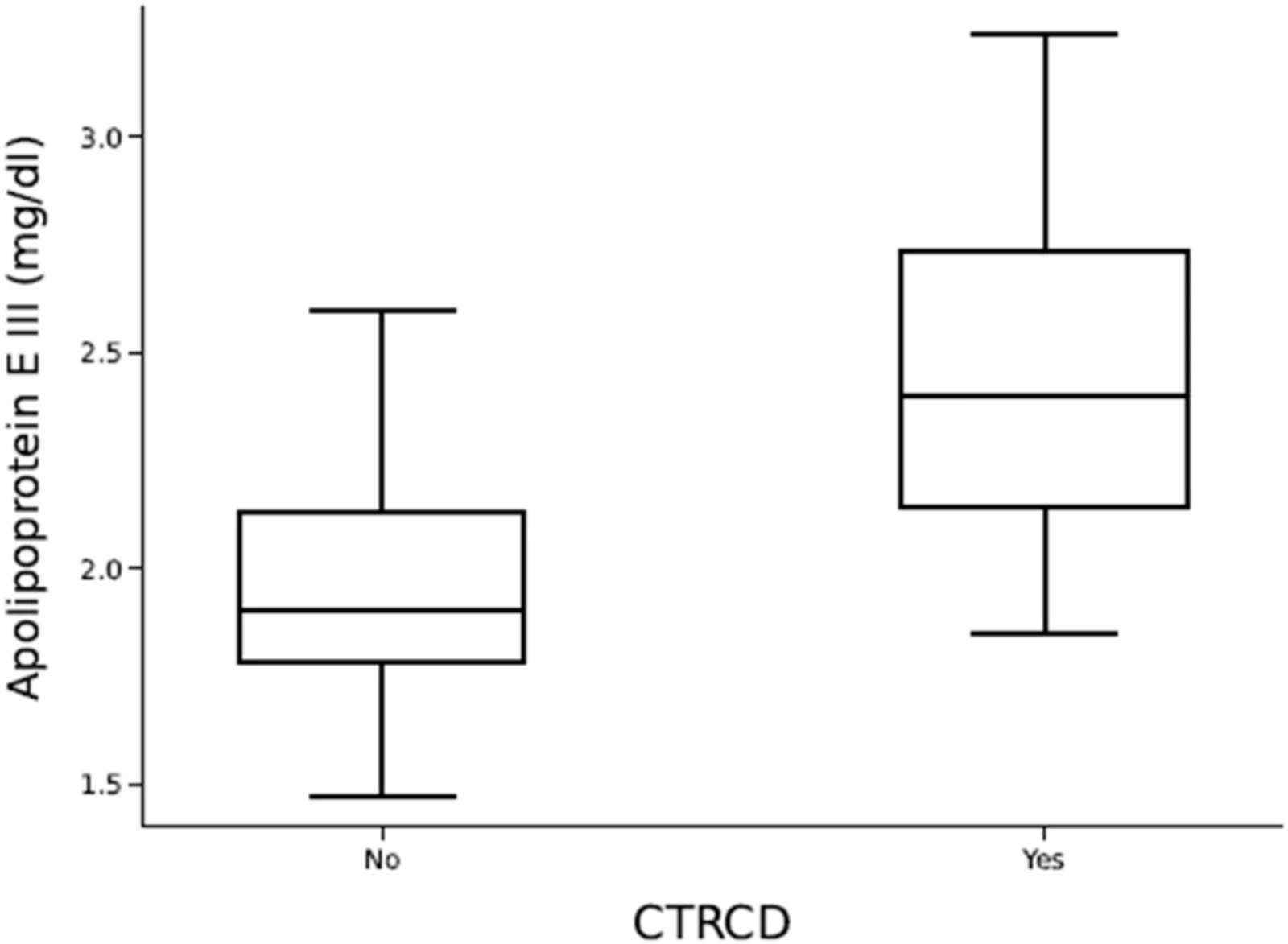
Comparison of apolipoprotein E concentrations at time point 3 (Apolipoprotein E III) between patients with cardiotoxicity (Yes) and without cardiotoxicity (No).

In addition to apolipoprotein C3, apolipoprotein E also warrants particular attention. Significant differences between patients with and without cardiotoxicity were observed at the 6-month follow-up after treatment completion (E III: 1.9 vs. 2.4 mg/dL; U = 25.0; *p* = 0.010), with higher ApoE concentrations observed in patients with cardiotoxicity (Figure 8).

To provide a reference point for the assessment of the significance of the analyzed parameters, the statistical significance of established markers of myocardial dysfunction, including hs-cTnT and NT-proBNP, was evaluated in the context of cardiotoxicity development. The analysis revealed significant differences in baseline NT-proBNP concentrations between patients with and without cardiotoxicity (U = 233; *p* = 0.027).

### 3.4. ROC Curve Analysis of Apolipoprotein Concentrations in Patients with and Without Cardiotoxicity

**Figure 9:**
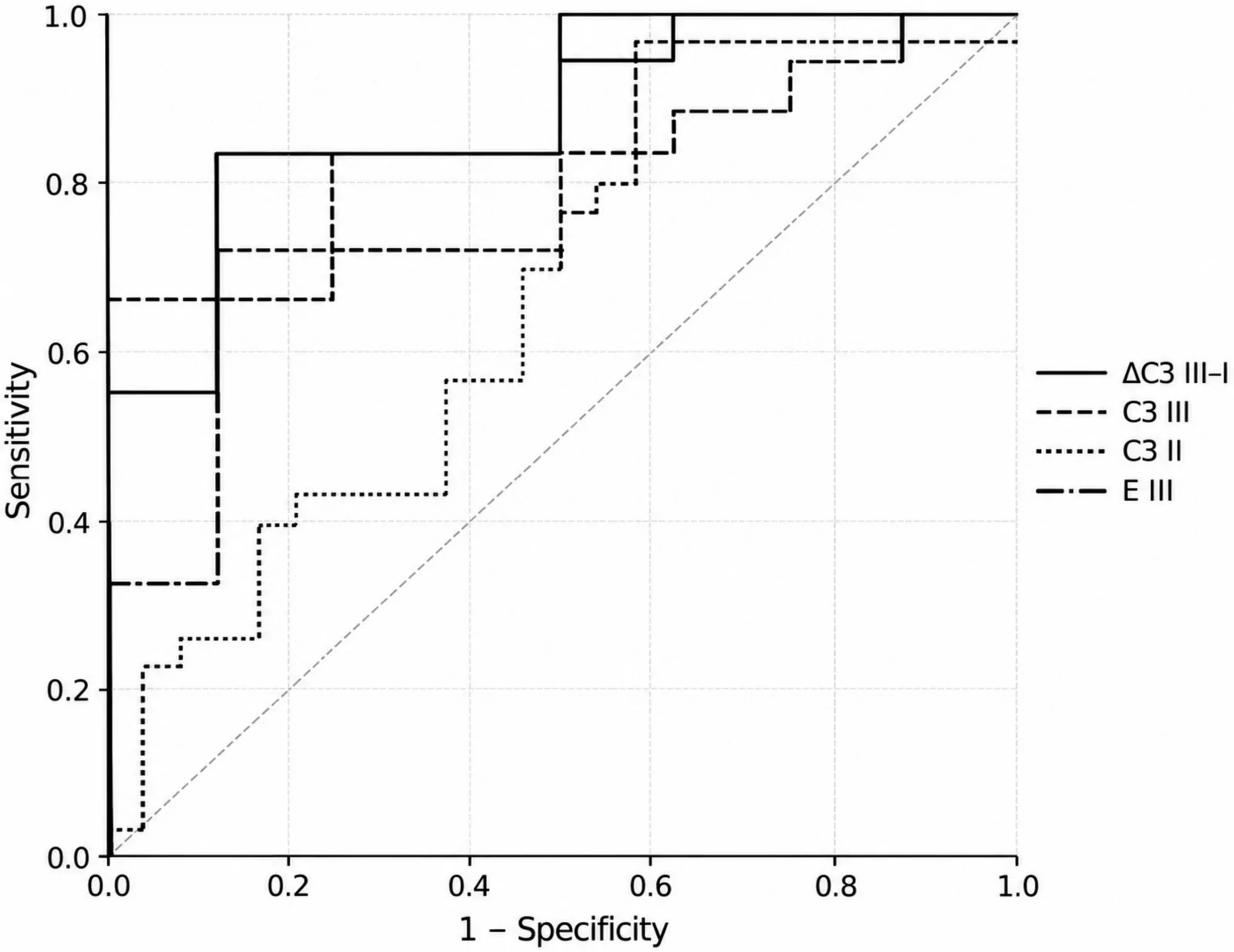
ROC curves of apolipoprotein E measured at time point 3 (E III), C3 measured at time point 2 (C3 II), at time point 3 (C3 III) and change in apolipoprotein C3 concentrations between time points 3 and 1 (ΔC3 III–I) for the development of cardiotoxicity (CTRCD).

ROC curve analysis was performed to assess the potential diagnostic value of apolipoproteins that were identified in previous analyses as being significantly associated with the development of CTRCD. The concentrations of apolipoprotein C3 measured immediately after treatment completion (C3 II) and at the 6-month follow-up after treatment completion (C3 III), as well as the difference between ApoC3 concentration at the 6-month follow-up and baseline concentration before treatment (ΔC3 III–I), were assessed. Additionally, the concentration of apolipoprotein E measured at the 6-month follow-up after treatment completion (E III) was evaluated. The analyzed parameters were assessed for their ability to discriminate between patients with cardiotoxicity (CTRCD = 1) and patients without cardiotoxicity (CTRCD = 0) (Figure 9).

The highest diagnostic performance was demonstrated by the change in ApoC3 concentration between baseline and the 6-month follow-up, with an AUC of 0.875 (95% CI: 0.735–1.000; *p* < 0.001). The optimal cut-off value, determined using the Youden index, was ≥31.50 mg/dL. At this threshold, the sensitivity was 83.3% and the specificity was 87.5%. The diagnostic accuracy was 84.6%, with a positive predictive value (PPV) of 93.8% and a negative predictive value (NPV) of 70.0%.

ApoC3 concentration measured at the 6-month follow-up after treatment completion (C3 III) demonstrated good discriminatory ability between patients with and without CTRCD, with an AUC of 0.833 (95% CI: 0.677–0.990; *p* < 0.001). For the optimal cut-off value of ≥46.02 mg/dL, the sensitivity was 66.7% and the specificity was 100.0%.

ApoC3 concentration measured immediately after treatment completion (C3 II) demonstrated moderate discriminatory ability between patients with and without CTRCD (AUC = 0.668; 95% CI: 0.518–0.818; *p* = 0.028). For the optimal cut-off value of ≥4.13 mg/dL, the sensitivity was 96.7% and the specificity was 41.7%.

Additionally, ROC analysis of apolipoprotein E measured at the 6-month follow-up after treatment completion (ApoE III) demonstrated good discriminatory ability between patients with and without CTRCD. The AUC value was 0.826 (95% CI: 0.643–1.000; *p* < 0.001). For the cut-off value of ≥2.18 mg/dL, the sensitivity was 72.2% and the specificity was 87.5%.

### 3.5. Assessment of Correlations Between Apolipoprotein and CRP Concentrations and Markers of Myocardial Injury

**Figure 10:**
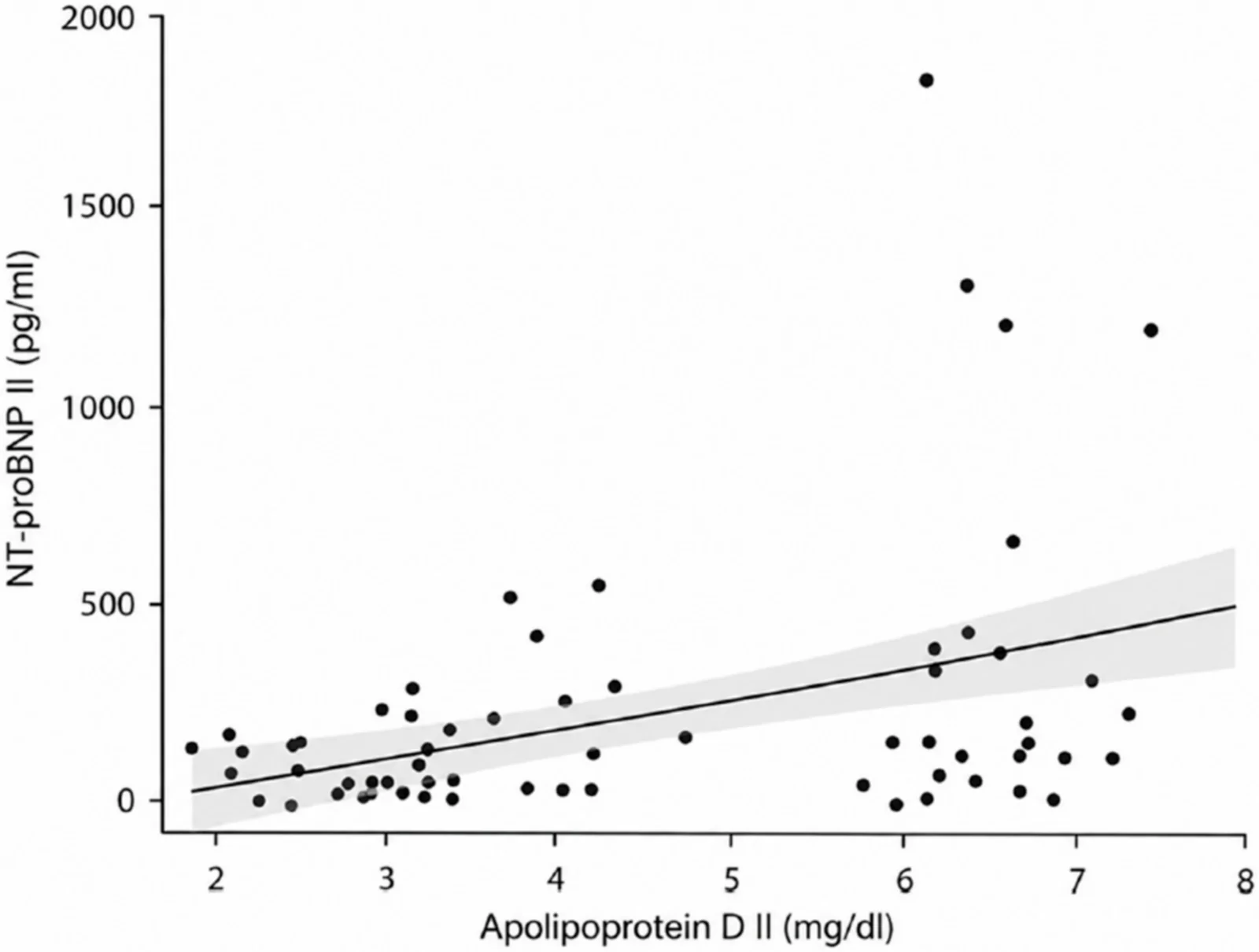
Scatter plot illustrating the relationship between apolipoprotein D concentration at time point 2 (Apolipoprotein D II) and NT-proBNP levels at time point 2 (NT-proBNP II).

**Figure 11:**
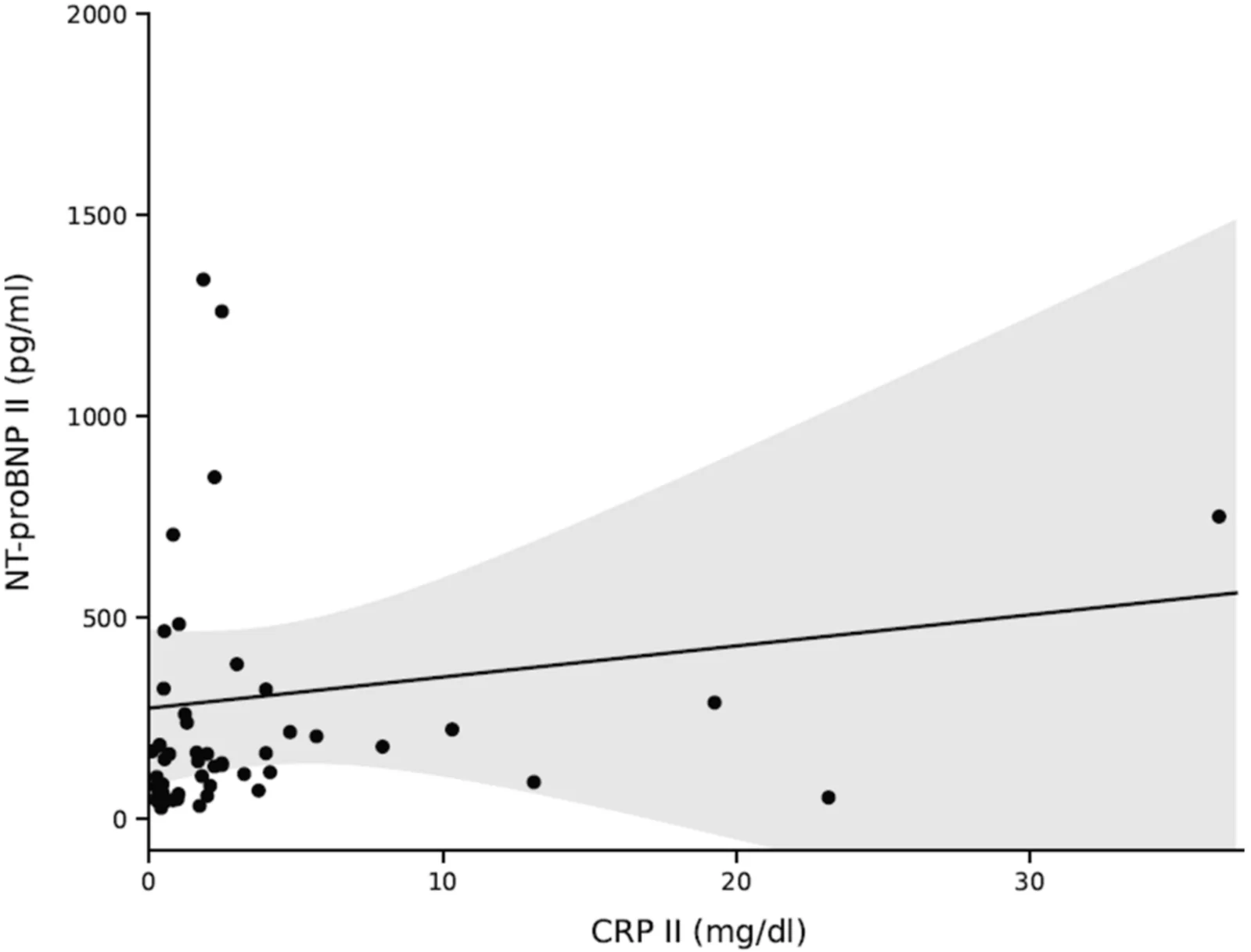
Scatter plot illustrating the relationship between C-reactive protein levels at time point 2 (CRP II) and NT-proBNP levels at time point 2 (NT-proBNP II).

The correlation analysis also revealed significant positive correlations between apolipoprotein D (ApoD) concentration and NT-proBNP concentration at the post-treatment time point (Spearman’s rho: R = 0.326; *p* = 0.015) and between CRP concentration and NT-proBNP concentration at the same time point (Spearman’s rho: R = 0.390; *p* = 0.003) (Figure 10 and Figure 11).

### 3.6. Logistic Regression Analysis Evaluating the Simultaneous Effects of Multiple Biomarkers on the Risk of Cardiotoxicity (CTRCD)

To assess the simultaneous impact of selected biomarkers on the risk of cardiotoxicity, a multivariable logistic regression analysis was performed. The model did not identify any statistically significant independent factors associated with CTRCD, which was most likely due to the limited sample size and the resulting insufficient statistical power.

## 4. Discussion

The aim of this study was to evaluate the association between concentrations of selected apolipoproteins and C-reactive protein and the occurrence of anthracycline-related cardiotoxicity in patients with breast cancer.

In this prospective study, we demonstrated that anthracycline treatment is associated with significant and persistent changes in the circulating apolipoprotein profile in patients with breast cancer. These changes were dynamic and heterogeneous: most of the analyzed apolipoproteins showed a gradual decrease in concentration, whereas ApoC3, ApoD, and CRP demonstrated an increasing trend during the follow-up period. Importantly, among all evaluated parameters, ApoC3, including the change in its concentration between baseline and the 6-month follow-up, as well as ApoE, showed the strongest association with the occurrence of treatment-induced cardiotoxicity (CTRCD). Furthermore, the dynamic increase in ApoC3 concentration demonstrated predictive value in ROC analysis, indicating its potential role as a candidate biomarker for the development of this complication.

The observed changes in circulating apolipoprotein concentrations during the study indicate that the effects of anthracyclines on these proteins are not limited to the period of active treatment but persist after completion of chemotherapy. The greatest differences were observed between the assessment immediately after treatment completion and the 6-month follow-up visit, suggesting that metabolic disturbances develop gradually and may reflect long-term consequences of anthracycline-induced injury. This observation is consistent with current knowledge regarding the pathogenesis of anthracycline cardiotoxicity, according to which myocardial injury is a prolonged, multifactorial process involving persistent oxidative stress, mitochondrial damage, disturbances in calcium homeostasis, endothelial dysfunction, and chronic activation of inflammatory pathways. These mechanisms contribute to progressive myocardial remodeling and disruption of multiple metabolic pathways, including lipid and lipoprotein metabolism. Previous studies have demonstrated that anthracyclines impair endothelial function and energy metabolism, which may indirectly affect the synthesis, transport, and catabolism of apolipoproteins [8,31,32].

Our findings are consistent with previous observations indicating that anthracycline-based anticancer therapy leads to disturbances in lipid metabolism and alterations in the concentrations of selected apolipoproteins. Li et al. demonstrated decreased ApoA1 concentrations in women treated for breast cancer, suggesting an adverse effect of chemotherapy on HDL lipoprotein homeostasis [33].

Sharma et al. observed changes in ApoA1 and ApoB concentrations and demonstrated that doxorubicin affects the expression of genes involved in lipid metabolism, including pathways responsible for HDL biosynthesis and transport [34].

In contrast to these studies, we simultaneously evaluated a broad panel of apolipoproteins and their changes at three consecutive time points, enabling a more comprehensive assessment of the metabolic response to anthracycline treatment.

An interesting observation was that the response of individual apolipoproteins was not uniform. Most of the analyzed proteins, including apolipoproteins A1, A2, B, C1, E, H, and J, demonstrated a gradual decrease in concentration, whereas ApoC3 and ApoD showed increasing concentrations over time. This suggests that individual apolipoproteins may reflect distinct biological processes activated during anthracycline treatment.

Some of the observed changes may result from disturbances in lipoprotein metabolism and alterations in hepatic protein synthesis. This interpretation is supported by a study demonstrating that anticancer treatment affects the expression of genes and the activity of pathways involved in lipid metabolism regulation, including PPARγ, LXRα, and ABCA1, leading to impaired HDL formation and transport as well as alterations in the metabolism of ApoB-containing lipoproteins [35].

The observed increase in ApoC3 and ApoD concentrations may reflect activation of inflammatory processes, oxidative stress, and adaptive responses to tissue injury, which is consistent with their documented roles in the regulation of inflammatory responses and cellular adaptation mechanisms [35,36,37,38].

However, it should be emphasized that not all observed changes in apolipoprotein concentrations appear to have the same clinical significance. Although anthracycline treatment affected the concentrations of most analyzed proteins, only a subset demonstrated an association with the development of cardiotoxicity. This suggests that the majority of observed changes reflect a systemic metabolic response to treatment, whereas only selected apolipoproteins may have potential value as biomarkers of myocardial injury. In our study, the most promising findings were obtained for ApoC3 and ApoE, whose potential roles in the development of CTRCD require further detailed investigation.

ApoC3 demonstrated the strongest association with the occurrence of cardiotoxicity. In our study, ApoC3 concentrations were significantly higher in patients with CTRCD both immediately after treatment completion and at the 6-month follow-up. Moreover, the dynamic change in ApoC3 concentration showed the highest diagnostic performance in ROC analysis. These findings suggest that monitoring changes in ApoC3 concentration over time may better reflect the development of myocardial injury than a single measurement of this biomarker. Importantly, in univariable analysis, ApoC3 consistently discriminated between patients with and without CTRCD, indicating its potential usefulness as a biomarker for monitoring the development of cardiotoxicity.

Available data regarding ApoC3 have so far focused primarily on its role in the metabolism of triglyceride-rich lipoproteins and the pathogenesis of cardiovascular disease. ApoC3 is currently recognized as one of the key regulators of triglyceride metabolism. By inhibiting lipoprotein lipase activity and hepatic uptake of triglyceride-rich lipoproteins, ApoC3 prolongs their residence time in plasma, promotes hypertriglyceridemia, and increases the atherogenic potential of these particles [39,40,41].

These findings have been confirmed and expanded by subsequent studies and recent reviews, indicating that ApoC3 not only regulates the metabolism of triglyceride-rich lipoproteins but also actively participates in inflammatory processes and atherogenesis. ApoC3 has been shown to exert direct pro-inflammatory effects through endothelial cell activation, increased expression of adhesion molecules such as VCAM-1 and ICAM-1, enhanced monocyte adhesion to the vascular wall, and activation of the NF-κB pathway, resulting in increased production of pro-inflammatory cytokines. Consequently, ApoC3 promotes endothelial dysfunction, chronic inflammation, and the progression of atherosclerosis [35,42,43,44,45].

These mechanisms may explain the association observed in our study between increased ApoC3 concentration and the development of CTRCD.

ApoE was the second apolipoprotein demonstrating a significant association with cardiotoxicity. Higher ApoE concentrations at the 6-month follow-up were observed in patients with CTRCD, and ROC analysis demonstrated good discriminatory ability for identifying this group. Although the diagnostic performance of ApoE was slightly lower than that of ApoC3, the obtained results suggest that this apolipoprotein may also represent a potential biomarker of processes associated with myocardial injury development.

Apolipoprotein E plays a key role in lipoprotein metabolism; however, its functions extend beyond lipid regulation. ApoE has been shown to participate in the modulation of inflammatory responses, regulation of endothelial function, and tissue repair processes following injury, highlighting its importance in maintaining cardiovascular homeostasis [45,46,47].

In the context of our study, the increase in ApoE concentration observed in patients with CTRCD may reflect the activation of endogenous protective mechanisms triggered in response to myocardial injury. This interpretation is consistent with the pleiotropic properties of ApoE described above, including its anti-inflammatory and anti-atherogenic effects and its involvement in tissue repair processes.

The ROC analysis results deserve particular attention, as the highest diagnostic performance was achieved by the change in ApoC3 concentration between baseline and the 6-month follow-up. This finding suggests that dynamic biomarker assessment may provide more information than a single measurement performed at one time point. This observation is consistent with current approaches to cardiotoxicity monitoring, which increasingly emphasize serial biomarker measurements and the assessment of their changes over time.

However, it should be emphasized that, to date, no studies have evaluated the significance of ApoC3 and ApoE as biomarkers of anthracycline-induced cardiotoxicity or directly linked changes in their concentrations with the development of CTRCD, highlighting the novelty of the present findings. Therefore, the proposed interpretation should be considered a biologically plausible hypothesis that requires confirmation in further prospective studies.

In the present study, most of the analyzed apolipoproteins did not demonstrate significant correlations with established markers of myocardial injury, including hs-cTnT and NT-proBNP. This finding suggests that apolipoproteins may reflect a different aspect of the pathophysiology of cardiotoxicity than commonly used cardiac biomarkers. While hs-cTnT is an established marker of cardiomyocyte injury and NT-proBNP reflects hemodynamic stress and developing myocardial dysfunction, changes in apolipoprotein concentrations may be associated with earlier disturbances in lipoprotein metabolism, inflammatory responses, oxidative stress, and endothelial dysfunction. These mechanisms play a key role in the pathogenesis of anthracycline-induced cardiotoxicity and may precede the development of overt myocardial injury detected by conventional biomarkers [4,8,32].

Two exceptions were significant positive correlations observed after treatment completion: between ApoD and NT-proBNP concentrations and between CRP and NT-proBNP concentrations. The positive correlation between ApoD and NT-proBNP may indicate the involvement of this apolipoprotein in the response to myocardial stress induced by anticancer treatment. ApoD is a glycoprotein belonging to the lipocalin family, involved in lipid transport and exhibiting antioxidant and cytoprotective properties. Experimental studies have demonstrated that ApoD expression increases under conditions of oxidative stress, ischemia, and tissue injury, suggesting its involvement in adaptive mechanisms that limit cellular damage. Therefore, the observed positive correlation with NT-proBNP may reflect an enhanced response to persistent cellular stress or cardiovascular burden after treatment completion [38,48].

It may be hypothesized that this association reflects the activation of adaptive mechanisms in response to subclinical myocardial injury. Due to the very limited number of studies investigating ApoD in cardio-oncology, this observation should be considered exploratory and requires confirmation in further studies. The observed positive correlation between CRP and NT-proBNP concentrations is consistent with current knowledge regarding the pathophysiology of anthracycline-induced cardiotoxicity, in which chronic activation of inflammatory pathways plays an important role in the development of myocardial injury. Increased CRP concentration may reflect persistent inflammation associated with processes leading to myocardial dysfunction, manifested by elevated NT-proBNP concentrations. This finding supports the concept that inflammatory processes may contribute to the development of early cardiovascular alterations associated with anthracycline treatment [4,11,49].

Despite the favorable results of univariable analyses and ROC analysis, multivariable analysis did not demonstrate an independent association between ApoC3, ΔC3 III–I, or ApoE and the risk of CTRCD development. The most likely explanation is the limited sample size, which substantially reduces the statistical power of logistic regression models. Therefore, the lack of statistical significance in multivariable analysis does not exclude the potential clinical relevance of these biomarkers but highlights the need for validation in larger cohorts.

The relatively small sample size also represents an important limitation of the present study, particularly for the ROC and subgroup analyses. The limited number of patients may result in an overestimation of the diagnostic performance of the investigated biomarkers. Therefore, the ROC-derived cut-off values should be considered preliminary and interpreted with caution. Their diagnostic value and clinical applicability require validation in larger, independent cohorts.

Another limitation of the present study is that no correction for multiple comparisons was performed, and the findings should therefore be considered exploratory. Given the preliminary, pilot nature of our study, the statistically significant findings require confirmation in larger patient cohorts. Further research is ongoing. Furthermore, the proportion of patients with CTRCD observed in our study should be interpreted in the context of the selected study cohort and should not be considered representative of the incidence of CTRCD in the general population.

In the present study, patients with HER2-positive breast cancer additionally received trastuzumab, which is known to have cardiotoxic effects. However, given the small proportion of patients receiving this treatment (2 patients, 4% of the study cohort), its overall contribution to the risk of cancer therapy-related cardiac dysfunction (CTRCD) and its potential influence on the observed associations between apolipoproteins and CTRCD were likely limited.

The obtained results indicate that anthracycline treatment leads to long-term changes in the apolipoprotein profile and CRP concentrations, reflecting a complex metabolic and inflammatory response to anticancer therapy. Among the evaluated biomarkers, apolipoprotein C3 demonstrated the greatest clinical potential, as its concentration and temporal changes during treatment enabled discrimination between patients with and without cardiotoxicity. Although these observations require validation in larger prospective studies, they suggest that serial assessment of ApoC3 may, in the future, represent a valuable complement to currently used strategies for monitoring anthracycline-related cardiotoxicity and may contribute to earlier identification of patients requiring more intensive cardiac surveillance.

Serial assessment of selected apolipoproteins, particularly the dynamics of changes in ApoC3 concentration, may represent a valuable addition to current strategies for monitoring CTRCD risk in cardio-oncology. However, further prospective studies involving larger patient populations are required to confirm the prognostic value of these biomarkers and to determine their potential clinical applicability.

Among the broad panel of analyzed apolipoproteins, apolipoprotein C3 and apolipoprotein E demonstrated potential relevance in the development of cancer therapy-related cardiotoxicity (CTRCD). A predictive value was observed for the dynamic increase in ApoC3 concentration, expressed as the difference between the concentration after treatment completion and the baseline concentration before treatment (ΔC3 III–I), suggesting that this parameter may serve as a potential marker supporting the identification of patients at increased risk of this complication.

## 5. Conclusions

An increase in apolipoprotein C3 concentration during treatment may have potential prognostic value for the development of cardiotoxicity, whereas measurement of apolipoprotein C3 and apolipoprotein E concentrations at the 6-month follow-up after completion of standard chemotherapy may enable discrimination between patients with breast cancer with diagnosed CTRCD and those without this complication.

## Figures and Tables

**Table 1:** Clinicopathological characteristics of patients.

Characteristic	Number of Patients (*n*)	Percentage (%)
Tumor stage (I–IV)		
I	26/54	48
II	20/54	37
III	6/54	11
IV	2/54	4
Tumor grade (G)		
G1	5/54	9
G2	29/54	54
G3	20/54	37
Estrogen receptors		
absence	16/54	30
presence	38/54	70
Progesterone receptors		
absence	17/54	31
presence	37/54	69
HER-2 status		
negative	42/54	78
positive	12/54	22
Ki-67 expression (cut-off 10%)		
low	10/54	18
high	44/54	82
Cardiovascular risk profile		
Diabetes mellitus		
no	46/54	85
yes	8/54	15
Hyperlipidemia		
no	15/54	28
yes	39/54	72
Statin therapy		
no	24/54	44
yes	30/54	56
Beta-blocker therapy		
no	31/54	57
yes	23/54	43
ACE inhibitor therapy		
no	30/54	56
yes	24/54	44
Cancer therapy-related cardiac dysfunction (CTRCD)		
no	24/54	44
yes	30/54	56

**Table 2:** Median (IQR) concentrations of apolipoproteins and C-reactive protein (CRP) at three study time points: baseline (I), immediately after completion of anthracycline therapy (II), and 6 months after treatment completion (III).

Marker	Median and IQR, mg/dL			Friedman Test, *p*-Value	Post-hoc Durbin–Conover test, *p*-Value ^1^
	Before Treatment (I)	After Treatment (II)	6 Months After Treatment (III)		
ApoA1	130.7 (80.4–138.5)	115.6 (57.3–120.9)	55.7 (46.9–65.7)	<0.001	<0.001
ApoA2	11.4 (10.4–90.6)	11.0 (6.7–11.8)	6.6 (5.6–8.5)	<0.001	<0.001
ApoB	41.6 (34.07–55.9)	48.9 (30.4–48.1)	17.2 (23.0–33.2)	<0.001	<0.001
ApoC1	44.1 (26.9–53.3)	54.8 (20.6–49.5)	18.5 (15.1–20.5)	<0.001	<0.001
ApoC3	9.9 (5.5–10.7)	10.9 (4.2–11.5)	45.3 (32.3–53.3)	<0.001	<0.001
ApoD	3.1 (2.9–6.7)	3.2 (2.9–6.2)	6.2 (6.0–6.7)	<0.001	<0.001
ApoE	4.0 (2.9–4.9)	4.2 (2.6–4.2)	2.3 (2.0–2.6)	<0.001	<0.001
ApoH	36.6 (28.8–42.7)	36.9 (22.8–38.6)	23.3 (19.9–26.3)	<0.001	<0.001
ApoJ	4.4 (3.3–5.0)	4.4 (2.7–4.4)	2.4 (2.2–2.7)	<0.001	<0.001
CRP	0.5 (0.3–0.7)	0.5 (0.5–3.4)	2.5 (1.6–3.8)	<0.001	<0.001

^1^ Post-hoc analysis presents *p*-values for pairwise comparisons between time points I and III and between time points II and III.

**Table 3:** Comparison of apolipoprotein concentrations between patients with and without cardiotoxicity immediately after completion of treatment (II) and 6 months after completion of treatment (III).

Apolipoprotein	Time Point	Median (IQR), mg/dL		U	*p*-Value
		No Cardiotoxicity	Cardiotoxicity		
A1	III	42.7 (36.1–55.4)	63.5 (50.9–67.0)	35.0	0.043
A2	III	5.6 (3.8–5.7)	7.6 (6.3–8.6)	27.0	0.013
C3	II	5.00 (3.7–10.2)	8.1 (5.0–13.4)	239.0	0.036
C3	III	33.5 (28.7–38.4)	47.8 (43.8–55.1)	24.0	0.006
D	III	6.0 (5.8–6.2)	6.4 (6.2–6.7)	34.0	0.037
E	II	4.3 (3.9–6.5)	3.8 (2.9–5.1)	236.0	0.032
E	III	1.9 (1.8–2.1)	2.4 (2.1–2.7)	25.0	0.010
H	III	21.3 (12.5–23.0)	24.2 (22.4–27.4)	35.5	0.045

## Data Availability

The data presented in this study are not publicly available due to privacy and ethical restrictions but are available from the corresponding author on reasonable request, subject to approval by the Institutional Review Board and applicable data protection regulations.

## Abbreviations

The following abbreviations are used in this manuscript:

ApoA1	apolipoprotein A1
ApoA2	apolipoprotein A2
ApoB	apolipoprotein B
ApoC1	apolipoprotein C1
ApoC3	apolipoprotein C3
ApoD	apolipoprotein D
ApoE	apolipoprotein E
ApoH	apolipoprotein H
ApoJ	apolipoprotein J
ApoM	apolipoprotein M
ACE-Is	angiotensin-converting enzyme inhibitors
AUC	area under the curve
CRP	C-reactive protein
CTRCD	cancer therapy-related cardiac dysfunction
DNA	deoxyribonucleic acid
ESC	European Society of Cardiology
GLS	global longitudinal strain
HDL	high-density lipoprotein
hs-cTnT	high-sensitivity cardiac troponin T
ICAM-1	intercellular adhesion molcule-1
IC-OS	International Cardio-Oncology Society
IDL	intermediate-density lipoprotein
IQR	interquartile range
LCAT	lecithin–cholesterol acyltransferase
LVEF	left ventricular ejection fraction
LDL	low-density lipoprotein
LXRα	liver X receptor alpha
NF-κB	nuclear factor kappa B
NPV	negative predictive value
NT-proBNP	N-terminal pro-B-type natriuretic peptide
PPARγ	peroxisome proliferator-activated receptor gamma
PPV	positive predictive value
RCT	reverse cholesterol transport
ROC	receiver operating characteristic
RNA	ribonucleic acid
ROS	reactive oxygen species
TNBC	triple negative breast cancer
TNM	tumor–node–metastasis classification
VCAM-1	vascular cell adhesion molecule-1
